# Measurement properties of the Dutch–Flemish patient-reported outcomes measurement information system (PROMIS) physical function item bank and instruments: a systematic review

**DOI:** 10.1186/s12955-020-01647-y

**Published:** 2021-02-24

**Authors:** Inger L. Abma, Bas J. D. Butje, Peter M. ten Klooster, Philip J. van der Wees

**Affiliations:** 1 Radboud University Medical Center, Radboud Institute of Health Sciences, IQ healthcare, Geert Grooteplein 21 (route 114), P.O. Box 9101, 6500 HB Nijmegen, The Netherlands; 2 Department of Psychology, Health, and Technology, Centre for eHealth and Well-Being Research, University of Twente, Enschede, The Netherlands

**Keywords:** Physical function, Instrument validation, PROMIS, Patient-reported outcome measure, Systematic review

## Abstract

**Background:**

Limitations in physical functioning are a big concern especially for patients with chronic or musculoskeletal diseases. Therefore, physical functioning is often used as a core outcome of treatments. The generic patient-reported outcomes information system (PROMIS) physical function (PF) item bank has shown potential to measure PF with better precision, interpretability and lower respondent burden compared with traditional patient-reported outcome measures. This study provides an overview of the current evidence on the quality of the measurement properties of the translated Dutch–Flemish PROMIS-PF item bank and its subdomains, and their derived short forms and computer adaptive tests (CATs).

**Methods:**

PubMed was searched up to June 17th 2020 for validation studies of Dutch–Flemish PROMIS-PF in Dutch and Flemish adults. Quality assessment of the included studies was conducted using the COSMIN Risk of bias checklist. The COSMIN criteria for good measurement properties were used to judge the results of the studies, which were adjusted and added to where needed for this review, in the context of IRT instruments and item banks. The quality of evidence was summarized for each measurement property based on the Grading of Recommendation Assessment, Development, and Evaluation (GRADE) approach.

**Results:**

Eleven studies were included, evaluating the PROMIS-PF item bank, the Upper Extremity (UE) subdomain, and/or their derived short forms and CATs in different clinical populations. There is evidence for sufficient structural validity, measurement precision, construct validity, and cross-cultural validity of the Dutch–Flemish PROMIS-PF item bank. The upper extremity subdomain item bank shows high quality evidence for structural validity and measurement precision. Content validity of these item banks has not been thoroughly demonstrated in a Dutch–Flemish population. Furthermore, the derived instruments have far less robust evidence: there are fewer validation studies available and none examined their performance as stand-alone administered instruments.

**Conclusions:**

The first studies into the Dutch–Flemish PROMIS-PF item bank and the UE subdomain show promising results, with especially high quality evidence for sufficient structural validity and measurement precision. However, more studies, and with higher methodological quality, are needed to study the instruments derived from these item banks. These studies should also evaluate content validity, reliability and responsiveness.

## Introduction

Limitations in physical functioning, or in the ability to perform (instrumental) activities of daily living [1], are a big concern especially for patients with chronic or musculoskeletal diseases [2]. Disability in physical functioning is often stated as a cause of dependency in daily life. Therefore, physical functioning should be seen as a core outcome of many treatments in these populations, which could be measured in clinical practice with patient-reported outcome measures (PROMs) [3, 4].

PROMs are questionnaires that can be used to measure patient-reported outcomes. In clinical practice, healthcare professionals use a variety of different PROMs to measure physical functioning, commonly consisting of a predefined set of validated questions. The scores on these different PROMs are not readily comparable, which limits for example the comparability of scores among different populations [5]. Moreover, there is often a lack of alignment between the interpretation of scores and clinical decision making by health care professionals [6]. Additionally, many PROMs have been reported to lack measurement precision and have a relatively high respondent burden [7, 8].

A promising way to overcome these problems with fixed-format PROMs is via computer adaptive testing [9]. A computer adaptive test (CAT) is a computer-administered measure with questions (items) that are selected by a computer algorithm, based on a patient’s response to previous items and their estimated health state within a specific health domain [9, 10]. The items in CATs originate from extensive item banks, which consist of a wide range of items that all measure the same construct [11]. Item banks are calibrated using Item Response Theory (IRT) analysis, which orders items from an item bank along a measurement continuum, based on their difficulty and discrimination ability [12]. In CATs, measurement precision can be optimized and floor and ceiling effects are minimized [13].

The Patient-Reported Outcomes Measurement Information System (PROMIS) project has developed and calibrated IRT item banks for assessing several important health domains, including physical function, across a wide variety of conditions for the United States (US) population [14]. PROMIS instruments have shown great potential with better interpretability, precision, and content validity as well as lower respondent burden compared with traditional PROMs [15–17]. For PROMIS instruments, each test results in a T-score, of which 50 is the average of the general population in which the item bank is calibrated, with a standard deviation of 10. Cut-off points for the severity of the T-scores are suggested for the different domains, making the results easily interpretable.

One of the item banks developed by PROMIS is the PROMIS-Physical Function (PF) item bank [18–23]. This item bank contains 121 items (v1.2), and can additionally be split into two subdomains: Mobility (44 items, v2.0) and Upper Extremity (UE) (46 items, v2.0). In addition to the possibility to use CATs of these item banks, several standard short forms (fixed sets of items) have been developed by PROMIS based on these item banks. These contain between 4 and 20 items that were selected to most reliably assess the construct.

In the Netherlands, the interest in using PROMIS to measure health outcomes has increased since its first implementation in the US. The PROMIS-PF item bank for adults was translated into Dutch in 2014 [24], and since then several validation studies of the Dutch–Flemish PROMIS-PF have been conducted in different patient groups. Recently, there has been increasing interest in the uptake of this item bank in Dutch clinical practice, where it could replace other (classical test theory (CTT) based) physical functioning PROMs. However, the implementation and maintenance of PROMIS CATs in clinical practice requires additional resources and investments [25]. In order to make a well-considered decision about whether PROMIS-PF could be used to replace other PROMs that measure physical functioning, an overview of the measurement properties of the item banks and derived instruments is essential. In this systematic review we therefore summarize the evidence on the measurement properties of the Dutch–Flemish PROMIS-PF item bank and its derived instruments (CATs and short forms), including the subdomains ‘Mobility’ and ‘Upper Extremity’.

## Methods

### The PROMIS-PF item bank

The PROMIS-PF item bank consists of items measuring self-reported capability to carry out activities that require physical actions, ranging from self-care (activities of daily living) to more complex activities that require a combination of skills, often within a social context. The item bank contains items concerning the functioning of the upper extremities (which form the UE subdomain), lower extremities (which form the Mobility subdomain), and central regions (neck, back), as well as activities of daily living, such as running errands [19]. There is no time frame for the items, but current status is inferred.

There are several versions of the PROMIS-PF item bank, with the latest being version 2.0 [26]. The only version translated into Dutch is v1.2 [24], which contains 121 items. A validation study of the UE subdomain has conducted a translation of four additional items to upgrade the subdomain to v2.0 [27].

An item bank is not the measurement instrument as it is administered to patients, but is the large set of calibrated items that feeds the actual instruments such as short forms or CATs. For the PROMIS-PF item bank, a set of fixed short forms has been developed based on the most informative items at the group level in the full item bank. These contain 20, 10, 8, 6 and 4 items, respectively. Additionally, CATs can be used in which items are selected for individual patients on an item-by-item basis. When using the standard PROMIS CAT stopping rules, patients will receive new questions until a certain level of precision of the score (standard error of ≤ 3) on the T-score metric is reached, with a maximum of 12 items. It is also possible to use a fixed-length CAT, in which each patient will be asked to complete a certain number of items: for example 4, 6, 8 or 10. Which items an individual patient is presented with is dependent on which question will be most informative considering their previous answer, as with any CAT. A patient that completes a PROMIS-PF CAT with 10 items (CAT-10) will have answered those items from the database that help estimate that patient’s score most precisely. This is different from a short-form, in which the items are fixed and presented all at once. When the short form PROMIS PF-10 and the CAT-10 are both completed by the same patient, the CAT-10 will likely give a more precise estimate of this patient’s level of functioning than the short-form.

The developers of the PROMIS item banks have only developed generic short-forms, but it is possible to develop tailored short-forms aimed at specific populations. Usually this is done by selecting items based on the relevance of their content from a clinical perspective or on their observed measurement performance on a specific level of the underlying metric.

The PROMIS-PF item bank and the UE subdomain are calibrated separately. Scores of short forms and CATs are based on the calibration of the item bank from which they are derived.

### Literature search

Pubmed was searched from inception up to June 17th 2020 for articles on validation studies about the measurement properties of Dutch–Flemish PROMIS-PF (CATs and short forms) in Dutch and Flemish adults. The key search elements included were: (1) PROMIS item bank (complete item bank and short forms), (2) physical functioning/upper extremity/mobility, (3) measurement properties (according to COnsensus-based Standards for the selection of health Measurement Instruments (COSMIN) guidelines [28]) and (4) Dutch population. These elements were combined using the operator ‘AND’. Moreover, an exclusion filter was added using the operator ‘NOT’ to exclude animal studies and irrelevant publication types [28]. The full search strategy can be found in Additional file 1. All remaining articles were screened for their relevance based on their title and abstract. Moreover, reference lists from the included articles were screened to identify additional articles, and the publication list of the website of the Dutch–Flemish PROMIS group (www.dutchflemishpromis.nl) was checked for any potentially missing studies.

### Selection criteria

Studies were included that evaluated the measurement properties of the Dutch–Flemish PROMIS-PF complete item bank, CAT or any (standard or newly developed) short forms in Dutch or Flemish adults (age ≥ 18). Studies were excluded if they did not evaluate (instruments derived from) the official translation of the PROMIS-PF item bank [24]. A first selection was made based on screening of title and abstract, followed by screening of the full texts. This was both done by two independent reviewers (IA and BB).

### Assessing measurement properties

In this review we use the terminology as determined by the COSMIN panel [29]. They divide the measurement properties into three domains [29]: (1) validity (including content validity, construct validity (i.e. structural validity, hypothesis testing, and cross-cultural validity)), (2) reliability (including internal consistency, (test–retest) reliability, and measurement error), and (3) responsiveness. The results for the measurement properties of each study were rated with standard criteria for good measurement properties as ‘sufficient’ (+), ‘indeterminate’ (?) or ‘insufficient’ (−) [28, 30, 31] (Table 1). Two reviewers (IA, and BB or PK) judged the measurement properties against the criteria and reached consensus.

**Table 1:** Criteria for good measurement properties^a^

Measurement property	Rating	Criteria
**Main category: validity**		
Content validity	+	≥ 85% of the items are relevant for the construct of interest, the target population, and the context of use AND no key concepts are missing (comprehensiveness) AND > 85% of items is comprehensible for the population of interest^b^
	?	Not all information for ‘+’ reported
	−	Criteria for ‘+ ’ not met
Structural validity^c^	+	**CTT** CFA: CFI or TLI or comparable measure > 0.95 OR RMSEA < 0.06 OR SRMR < 0.08
		**IRT/Rasch** No violation of unidimensionality: CFI or TLI or comparable measure > 0.95 OR RMSEA < 0.06 OR SRMR < 0.08 *OR (for item banks only)* *Bifactor model: Standardized loadings on common factor (H) are* > *0.30 and larger than loadings on group factors OR high coefficient omega (*> *0.80) and a high ECV (*> *0.60)* *AND (for item banks: OR)* No or limited violation of local independence: Residual correlations among the items after controlling for the dominant factor < 0.20 in ≥ 95% of item pairs OR in < 95% of item pairs but evidence shown that impact is negligible OR Q3′s < 0.37 *AND* No violation of monotonicity: Adequate looking graphs OR item scalability (Hi) > 0.30 *AND (not for item banks)* Adequate model fit IRT: χ^2^ *p*-value > 0.001 Rasch: infit and outfit mean squares ≥ 0.5 and ≤ 1.5 OR Z-standardized values > − 2 and < 2
	?	Not all information for ‘+’ reported OR residual correlations among the items after controlling for the dominant factor < 0.20 in < 95% of item pairs but no evidence shown on the impact
	−	Criteria for ‘+’ not met
Hypothesis testing for construct validity	+	Result is in accordance with hypothesis^d^
	?	No hypothesis defined (by the review team)
	−	Result is not in accordance with hypothesis^d^
Cross-cultural validity/measurement invariance	+	No important differences found between group factors (such as age, gender, language) in multiple group factor analysis OR DIF in ≤ 5% of item pairs for group factors (e.g., McFadden’s R^2^ < 0.02) OR DIF in > 5% of item pairs but evidence shown that impact is negligible
	?	No multiple group factor analysis OR DIF analysis performed, OR DIF in > 5% of item pairs and no evidence shown on impact
	−	Important differences between group factors OR DIF was found in > 5% of item pairs with no mention of impact or evidence showing that impact is not negligible
**Main category: Reliability**		
Internal consistency/measurement precision	+	**CTT** At least low evidence^e^ for sufficient structural validity^f^ AND Cronbach’s alpha(s) ≥ 0.70 for each unidimensional scale or subscale **IRT** At least low evidence^e^ for sufficient structural validity^f^ AND reliability coefficient ≥ 0.90 over a range of at least two standard deviations around the average of the study population (or ≥ 68% of the study population)
	?	Criteria for “At least low evidence^e^ for sufficient structural validity^f^” not met
	−	Criteria for “At least low evidence^e^ for sufficient structural validity^f^^”^ AND other criteria for + not met
Reliability	+	ICC or weighted Kappa ≥ 0.70
	?	ICC or weighted Kappa not reported
	−	ICC or weighted Kappa < 0.70
Measurement error	+	SDC or LoA < MIC^e^
	?	MIC not defined
	−	SDC or LoA > MIC^e^
**Main category: Responsiveness**		
Responsiveness	+	Result is in accordance with hypothesis^d^ OR AUC ≥ 0.70
	?	No hypothesis defined (by the review team)
	−	Result is not in accordance with hypothesis^d^ OR AUC < 0.70

*AUC,* area under the curve; *CFA,* confirmatory factor analysis; *CFI,* comparative fit index; *CTT,* classical test theory; *DIF,* differential item functioning; ECV, explained common variance; *ICC,* intraclass correlation coefficient; *IRT,* item response theory; *LoA,* limits of agreement; *MIC,* minimal important change; *RMSEA,* root mean square error of approximation; *SEM,* standard error of measurement; *SDC,* smallest detectable change; *SRMR,* standardized root mean residuals; *TLI,* Tucker–Lewis index

“ + ” = sufficient, “?” = indeterminate, “ − ” = insufficient

^a^Adjusted from the COSMIN criteria [30, 31] as described in the “Methods” section

^b^From the COSMIN guidelines on evaluating content validity [30]

^c^Structural validity is not relevant for CATs

^d^The results of all studies taken together should show that 75% of the results are in accordance with the hypotheses [31]

^e^As defined by grading the evidence according to the GRADE approach

^f^This evidence may come from a different study

The COSMIN taxonomy and criteria for study quality and measurement properties have been specifically developed for traditional, fixed-length measurement instruments and are mostly focused on CTT criteria. Therefore, we considered some general additions necessary for instruments evaluated with IRT. Additionally, in the context of evaluating IRT-based *item banks* [32], some important differences arise, since item banks are not the actual instruments (short forms or CATs) that will be used in studies and daily care, but the pool of items feeding such instruments. Some measurement properties (test–retest reliability and responsiveness) are therefore not relevant or practically feasible for evaluating an item bank at all. For other measurement properties, we made changes or additions as necessary, a practice that is encouraged in the COSMIN manual [28]. Furthermore, when it comes to CATs, structural validity is not feasible to determine because different items are presented in each test. Information on structural validity should be gathered from the results for the item bank on which the CAT is based. All changes made in the COSMIN criteria are explained below.

The statistical approach and procedures of testing the measurement properties of an IRT-based instrument generally differ from those used to develop and evaluate CTT-based instruments. For instance, structural validity in CTT is usually tested via factor analysis only. In developing or evaluating IRT-based item banks, unidimensionality is also frequently tested via traditional exploratory or confirmatory factor analysis (FA), but also increasingly via additional models such as bifactor modeling. Since COSMIN does not propose criteria for the results of a bifactor model, we added criteria for demonstrating essential unidimensionality of an item bank based on the literature: standardized loadings on the common factor > 0.30 and larger than loadings on group factors (criterion proposed by the developers of PROMIS [10]) or a high coefficient omega (> 0.80) and a high explained common variance (ECV; > 0.60) [33, 34]. In addition to criteria for unidimensionality, COSMIN also proposes criteria for local independence, monotonicity and adequate model fit for IRT-based instruments. The separate criteria for assessment of adequate model fit (e.g., χ^2^ *p*-value > 0.001) were disregarded for item banks, because the appropriateness of this specific statistic and cut-off for significance is unclear for item banks and is rarely mentioned in their validation articles. Additionally, for item banks, the criterion for local independence (no local dependence allowed) may be considered too strict considering the large pool of items. We therefore adjusted this criterion to: ≤ 5% of item pairs show local dependence, or if this percentage is higher, evidence is shown that the impact of local dependence on item parameters or ability estimates is negligible. The same adjustment was made for cross-cultural validity/measurement invariance with regard to percentage of items with differential item functioning (DIF) and their impact.

In CTT, internal consistency is assessed via a global indicator of reliability, such as Cronbach’s alpha. IRT additionally allows the assessment of local reliability or measurement precision along the underlying scale by means of test information values, which can be recalculated to show standard errors or r-values across different levels of theta (IRT-based score) [35]. Since there is no criterion proposed by Terwee et al. [32] for internal consistency within an IRT context, in this review internal consistency was judged sufficient when the local reliability coefficient is ≥ 0.90 over a range of at least two standard deviations around the average of the study population. For example, theta − 1 and 1 if analysis is performed in the same population in which the item bank was calibrated. A criterion of ≥ 0.90 is commonly used when studying individual-level applications of an instrument [36, 37].

### Assessing methodological quality

Quality assessment of the included studies was conducted using the COSMIN Risk of bias checklist and scoring system [28, 30, 38] by two independent reviewers (IA and BB). This standardized checklist consists of nine boxes with items stating how each measurement property should be assessed, and an additional box about PROM development that can be taken into account when evaluating content validity. For each included study, the relevant boxes were rated as ‘very good’, ‘adequate’, ‘doubtful’ or ‘inadequate’ quality. Consistent with the COSMIN procedures, the lowest item score of each box was used to determine the overall score of its corresponding measurement property [39]. Since there is no gold standard for physical functioning, the measurement property ‘criterion validity’ is not assessed in this article. Studies in which PROMIS-PF is compared to existing legacy instruments for physical function are considered to assess construct validity, as per the COSMIN guidelines.

Often, terms and definitions used in papers to describe a measurement property were not consistent with COSMIN. In such cases, the COSMIN taxonomy was applied to determine which property was being reported [29]. Development of new short forms for specific patient groups were considered ‘modifications’ of the item bank rather than completely new instruments [30]. The COSMIN box ‘content validity’ rather than ‘instrument development’ is therefore scored for these development studies.

### Synthesis of the evidence

The quality of evidence was summarized for each measurement property based on a modified version of the Grading of Recommendation Assessment, Development, and Evaluation (GRADE) approach for systematic reviews [40]. As per the instructions of the COSMIN guidelines, the following factors of the GRADE approach to determine the quality of evidence were taken into account: (1) risk of bias (i.e. the methodological quality of the studies), (2) inconsistency (i.e. inconsistent results across studies unexplained), (3) imprecision (i.e. total sample size of the available studies), and (4) indirectness (i.e. provided evidence across different populations besides those of interest in the review). The GRADE approach assumes the overall evidence/result of the measurement properties is of ‘high’ quality. However, the quality can be downgraded by one or two levels to ‘moderate’ or ‘low’ quality of evidence, depending on the seriousness of risk of bias, inconsistency, imprecision or indirect results. Additionally, the quality of evidence can also be graded as ‘very low’ when the evidence was based on one inadequate study only (with extremely serious risk of bias). More detailed information on the interpretation and application of the four GRADE factors in evaluating the quality of evidence can be found in the COSMIN guideline [28].

For some measurement properties, the results can potentially be statistically pooled: internal consistency (if calculated by global indices such as Cronbach’s alpha, not for IRT-based local measurement precision), (test–retest) reliability, measurement error, hypotheses testing for construct validity, and responsiveness. For this review, results were pooled if this was both possible and relevant for the summary of the evidence according to the GRADE approach. The following criteria were used for when pooling was relevant: (1) if the sample size of one of the studies was below 100, since a smaller sample size requires downgrading of the summarized evidence according to the GRADE approach of COSMIN, or (2) if pooling would have an impact on the overall conclusion of the quality of the measurement property (e.g. if two studies find consistent results meeting the criteria for that measurement property, pooling of the results will not add new information or change the conclusion; however, when the result is indeterminate because two studies find results that do and do not meet the criteria (inconsistent results), pooling can be used to reach a conclusion).

For hypothesis testing, 75% of the results of all studies taken together should be consistent with hypotheses. This is done by adding up the total number of (confirmed and unconfirmed) hypotheses. Additionally, the correlations underlying the hypotheses can potentially be statistically pooled if two studies report correlations between the same instruments. This was only considered in this review if the abovementioned criteria were met.

Lastly, regarding content validity, we have considered that some results may ‘extrapolated’ from an item bank to its instruments. The comprehensibility and relevance of the (items of) the short forms and CATs can potentially be derived from studies into the full item bank. The overall COSMIN rule remains that 85% of items should be comprehensible/relevant, therefore the quality of the measurement property for the item bank may differ from that of a specific short form (e.g. if a total of 3 items of an item bank with 100 items are not relevant then the item bank has sufficient relevance; if these 3 items are all in a 4-item short form then the short form has insufficient relevance). For this review, we have considered comprehensibility suitable to be extrapolated across all populations; for relevance this may differ per population (e.g. running 20 miles may not be relevant for elderly people).

## Results

### Literature search and characteristics of included studies

**Fig. 1:**
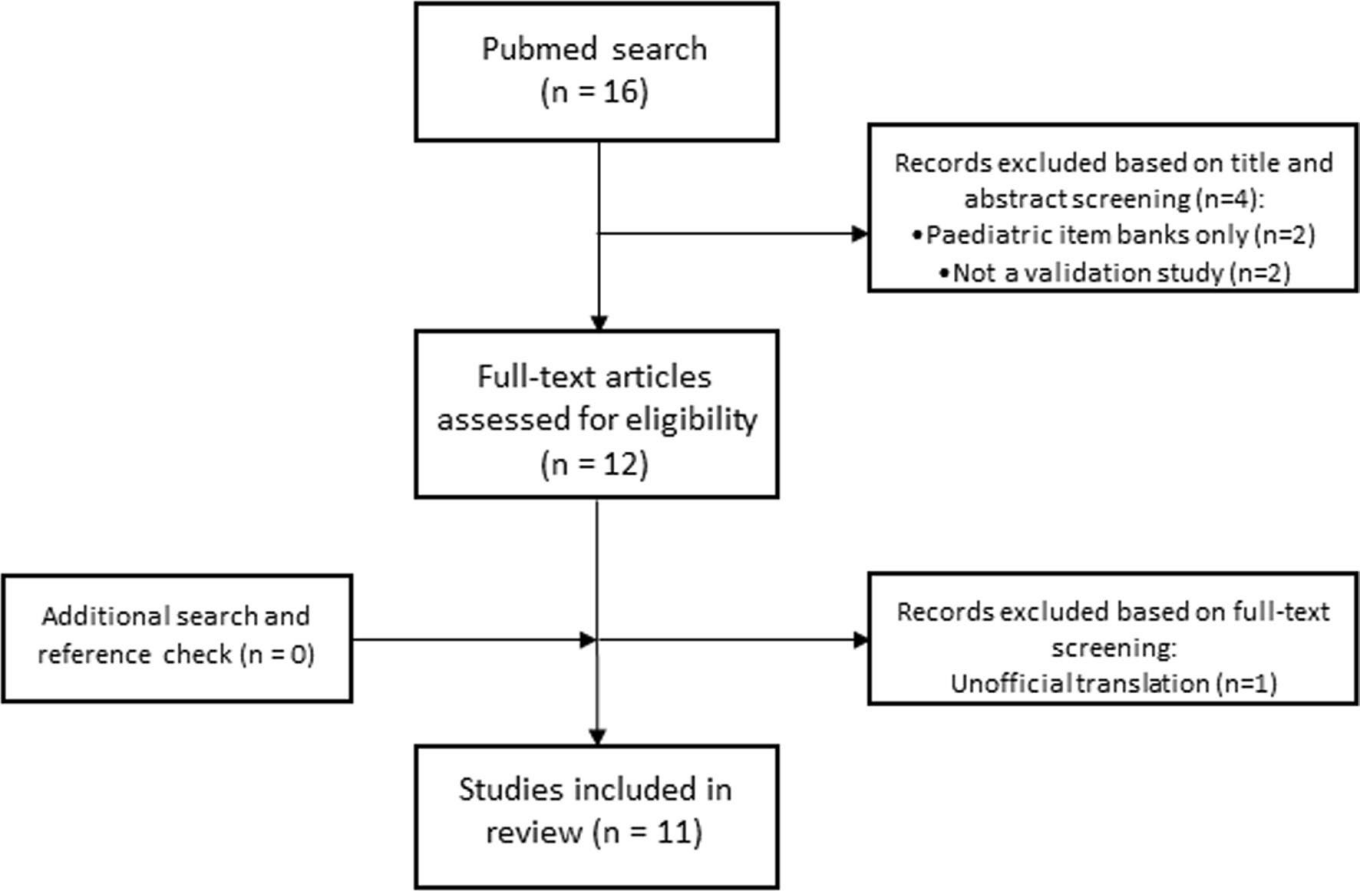
Flow chart of literature search and article selection

The literature search in PubMed identified 16 records of which 11 were deemed eligible for this study [24, 27, 36, 37, 41–48]. Four studies were excluded after title and abstract screening, one after full-text screening (Fig. 1). Reasons for exclusion were the assessment of the validity of PROMIS-PF in children only, the study was not a validation study, or an unofficial translation of the PROMIS-PF item bank was studied.

The characteristics of the included studies are shown in Table 2. Apart from the translation study [24], which had a study population which was both Dutch and Flemish, all studies were conducted in a Dutch population only. Six studies [24, 37, 41–44] evaluated the general Dutch–Flemish PROMIS-PF item bank, with or without additionally assessing CATs and its standard short form(s). One study only assessed the standard short forms [36]. The studies took place in a variety of populations: the general population, patients with rheumatoid arthritis (RA), patients with (chronic) musculoskeletal pain complaints, patients with osteoarthritis, and patients receiving physiotherapy. Additionally, two studies developed and assessed short forms for specific patient groups: one for patients with RA (PROMIS-PF-RA; 20 items) [37], and one for geriatric rehabilitation patients (PROMIS-PF-GR; 24 items) [47].

**Table 2:** Characteristics of the included studies

References	n	Age	Sex	PROMIS-PF instruments	Study population	Measurement properties	Aim
		Mean (SD)	% male				
Terwee et al. [24]	70	49	42	PROMIS-PF item bank^a^	Dutch and Flemish general population	Content validity	Translate 17 PROMIS item banks for adults from English into Dutch–Flemish and perform a pilot test
Oude Voshaar et al. [41]	690	57 (12)	36	PROMIS-PF item bank^a^	Dutch patients with RA	Structural validity Cross-cultural validity	Calibrate PROMIS-PF in RA patients and to evaluate cross-cultural validity
Oude Voshaar et al. [37]	690	57 (12)	36	PROMIS-PF item bank^a^ PROMIS-PF-RA^b^	Dutch patients with RA	Content validity Construct validity Measurement precision	Evaluate the content validity and measurement properties of PROMIS-PF in patients with RA and develop a short-form for patients with RA
Crins et al. [42]	1247	48 (13)	22	PROMIS-PF item bank^a^ PROMIS-PF-20, 10, 8, 6, 4^c^ CAT-10, 8, 6, 4^c^	Dutch adults who had at least one chronic pain condition of the musculoskeletal system for at least 3 months	Structural validity Construct validity Cross-cultural validity Measurement precision	Assess the psychometric properties of PROMIS-PF in Dutch patients with chronic pain
Crins et al. [43]	805	53 (14)	41	PROMIS-PF item bank^a^ PROMIS-PF-20, 10, 8, 6, 4^c^	Dutch adults who received physical therapy in primary care in the past year	Structural validity Construct validity Cross-cultural validity Measurement precision	Assess the psychometric properties of PROMIS-PF in Dutch patients receiving physical therapy
Crins et al. [44]	1247	48 (13)	22	PROMIS-PF item bank^a^	Dutch adults with musculoskeletal pain	Cross-cultural validity	Assess cross-cultural validity of PROMIS-PF, -PI, -PB in Dutch patients with chronic pain
	1247	69 (8)	23	PROMIS-PF-20^c^	Dutch adults with osteoarthritis		
	805	53 (14)	41		Dutch adults receiving physical therapy		
	1310	51 (17)	47		Dutch general population		
Chiarotto et al. [36]	768	49 (13)	23	PROMIS-PF-20, 10, 8, 6, 4^c^	Dutch adults with musculoskeletal pain complaints for at least 3 months	Structural validity Construct validity Measurement precision	Assess measurement properties of PROMIS-PF short forms in patients with chronic low back pain
van Bruggen et al. [45]	303	50 (18)	52	Upper Extremity subdomain^d^ PROMIS-UE-7a	Dutch adults with an injury of the upper extremity	Structural validity Construct validity Measurement precision	Validate PROMIS-PF—Upper Extremity subdomain in Dutch patients
Haan et al. [27]	218	53	50	Upper extremity subdomain^d^	Dutch patients with musculoskeletal upper extremity disorders	Content validity Cross-cultural validity Construct validity	Validate PROMIS-PF—Upper extremity subdomain in Dutch patients
Lameijer et al. [46]	521	51 (17)	49	Upper extremity subdomain^d^ UE CAT^c^ UE CAT-7^c^	Dutch patients with musculoskeletal upper extremity disorders and patients with an injury of the upper extremity (pooled data from Van Bruggen et al. and Haan et al. )	Structural validity Measurement invariance Cross-cultural validity/measurement precision	Studying the model fit and measurement precision of the PROMIS-PF – Upper extremity subdomain
Smit et al. [47]	207	80 (8.3)	42	PROMIS-PF-GR^e^	Dutch geriatric rehabilitation patients	Content validity Structural validity Measurement invariance Internal consistency	Development and validation of a short form for measuring physical function in geriatric rehabilitation patients

*CAT-[number],* computer adaptive test with [predefined number] of items; PB*,* pain behavior; PF, Physical Function; PI*,* pain interference; PROMIS-PF-[number], short form with [number] items; RA, rheumatoid arthritis; UE—upper extremity

^a^Physical functioning item bank version 1.2

^b^Short form developed for RA patients (20 items)

^c^These instruments were not distributed to patients separately, but rather the data collected from the complete item bank administration was used to determine the quality of the measurement properties

^d^Upper extremity subdomain version 2.0

^e^Short form developed for geriatric rehabilitation patients (24 items)

Three of the studies evaluated measurement properties of the Dutch–Flemish PROMIS-PF UE subdomain item bank [27, 45, 46], two of which also studied the UE short form 7a, and one of which also studied the UE CAT with 7 items and a UE CAT with standard stopping rules (standard error of 3 on the T-score metric is reached, maximum of 12 items). These studies took place in patients with an injury or disorder of the upper extremity. The study of Lameijer et al. [46] used pooled data from the other two studies. No studies were identified that studied the measurement properties of the Mobility subdomain of the Dutch–Flemish PROMIS-PF item bank.

Most measurement properties, for the item banks and the short forms/CATs, were assessed in at least one study. However, none of the studies in this review assessed responsiveness, and only internal consistency/measurement precision was assessed as a measure for reliability.

### Methodological quality

The methodological quality of the studies is summarised in Table 3 for the studied measurement properties of the PROMIS-PF item bank and the UE subdomain item bank, and in Table 4 for the studied measurement properties of the instruments derived from these item banks.

**Table 3:** Methodological quality of studies assessing the PROMIS-PF item bank and subdomains per measurement property

References	Study population	Item bank	Content validity	Structural validity	Hypothesis testing		Cross-cultural validity/measurement invariance	Internal consistency/measurement precision
			Score	Score	(Convergent) Score	(Known-groups) Score	Score	Score
Terwee et al. [24]	General population	*PF item bank*	Doubtful					
Oude Voshaar et al. [41]	RA patients	*PF item bank*					Doubtful	
Oude Voshaar et al. [37]	RA patients	*PF item bank*	Doubtful		Adequate	Doubtful		Very good
Crins et al. [42]	Patients with chronic pain	*PF item bank*		Very good	Very good		Inadequate (CC validity)/Doubtful (MI)	Very good
Crins et al. [43]	Physical therapy patients	*PF item bank*		Adequate	Very good		Doubtful	Very good
Crins et al. [44]	Chronic pain; OA; Physical therapy; General population	*PF item bank*					Inadequate	
van Bruggen et al. [45]	Upper extremity injury	*UE subdomain*		Very good	Very good			Inadequate
Haan et al. [27]	Upper extremity disorder	*UE subdomain*	Doubtful		Very good		Inadequate	
Lameijer et al. [46]	Upper extremity disorder	*UE subdomain*		Very good (for FA)/Adequate (for bifactor model)			Doubtful	Very good

CC validity, cross-cultural validity; OA, osteoarthritis; FA, factor analysis; MI, measurement invariance; PF, physical function; RA, rheumatoid arthritis; UE, upper extremity

**Table 4:** Methodological quality of studies assessing short forms and CATs, per measurement property

References	Study population	PROMIS-PF instruments	Content validity	Structural validity	Hypothesis testing		Cross-cultural validity/measurement invariance	Internal consistency/measurement precision
			Score	Score	(Convergent) Score	(Known-groups) Score	Score	Score
Oude Voshaar et al. [37]	RA patients	*PROMIS-PF-RA*	Doubtful					Doubtful
Crins et al. [42]	Patients with chronic pain	*PROMIS-PF-20,10,8,6,4* *CAT-10,8,6,4*						Doubtful
Crins et al. [43]	Physical therapy patients	*PROMIS-PF-4,6,8,10,20*						Doubtful
Crins et al. [44]	Chronic pain; OA; Physical therapy; General population	*PROMIS-PF-20*					Doubtful	
Chiarotto et al. [36]	Muskoskeletal pain complaints	*PROMIS-PF-4,6,8,10,20*		Doubtful	Doubtful	Doubtful		Doubtful
van Bruggen et al. [45]	Upper extremity injury	*PROMIS-UE-7a*						Inadequate
Lameijer et al. [46]	Upper extremity disorder and injury	*PROMIS-UE-7a UE CAT*^*a*^ *UE CAT-7*^*b*^						Doubtful
Smit et al. [47]	Geriatric rehabilitation patients	*PROMIS-PF-GR (24 items)*	Doubtful	Very good (for FA)/Inadequate (for IRT analyses)			Very good	Inadequate

CAT-[number], computer adaptive test with fixed [number] of items; FA, factor analysis; GR, geriatric rehabilitation; IRT, item response theory; OA, osteoarthritis; PF, physical function; PROMIS-PF-[number], short-form with[number] of items; RA, rheumatoid arthritis; UE, upper extremity

^a^With standard PROMIS CAT stopping rules (standard error of 3 on the T-score metric is reached, maximum of 12 items)

With the exception of one study by Smit et al. [47], all studies into the measurement properties of the short forms and CATs did not administer these instruments as such, but rather used the data collected from the complete item bank administration. In other words, patients completed (large sections of) the full item bank, and to study the measurement properties of a short form only the data from the relevant items were selected and analysed. For CATs, simulated CATs were also created based on the patients’ answers to the full item bank. For both short forms and CATs this is considered a minor methodological flaw because it may bias results: patients may respond differently (e.g., as a result of ordering or fatigue effects) if they complete 10 items rather than a large set of items. This means that the methodological quality rating of these studies cannot be higher than ‘doubtful’ when scoring the COSMIN checklist. Only for content validity we did not need to downgrade for this flaw because this is measurement property was studied by different methods. All studies that assessed (aspects of) content validity scored ‘doubtful’, either because content validity was not assessed via (focus group) interviews or because it was unclear if the interviews were recorded and how they were analysed. Scores for structural validity ranged from ‘inadequate’ to ‘very good’. Scores lower than ‘very good’ for this measurement property were linked to the number of respondents, which for an optimal IRT analysis needs to be very high, and/or the abovementioned methodological flaw regarding the short forms and CATs being based on complete item bank administration. Hypothesis testing was scored ‘very good’ in many studies. The ‘known-groups’ approach had two ‘doubtful’ scores because important characteristics of the compared groups were not described. Cross-cultural validity/measurement invariance had generally low scores for methodological quality either because it was not clear whether the compared groups differed regarding relevant characteristics, or because it was clear that there were differences. For internal consistency/measurement precision scores ranged from ‘inadequate’ to ‘very good’. The ‘inadequate’ scores were due to the calculation of Cronbach’s alpha rather than local IRT-based measures. The ‘doubtful’ scores were due to uncertainty around the structural validity of several short forms, as this was not studied, and/or the abovementioned methodological flaw regarding the short forms and CATs.

### Quality of the measurement properties

The quality of the measurement properties of the item banks (PROMIS-PF item bank and the UE subdomain) can be found in Table 5, and the quality of the measurement properties of the instruments in Table 6. A summary of the evidence for both item banks and instruments can be found in Table 7. No statistical pooling was performed for any of the measurement properties: either the measured parameters could not be statistically pooled or statistical pooling would have had no impact on the overall (pooled) summary of the evidence with the GRADE approach, as explained in “Synthesis of the evidence” section.

**Table 5:** Quality of measurement properties and summary of the evidence for the item bank and subdomains

References	PROMIS-PF instrument	Population	Score	Description
*Content validity*				
Terwee et al. [24]	PF item bank	General population	?	Relevance and comprehensiveness not studied
			+	Sufficient comprehensibility
Oude Voshaar et al. [37]	PF item bank	RA patients	+	Sufficient relevance and comprehensiveness based on linking the item bank to the ICF core set for RA
			?	Comprehensibility not studied
Haan et al. [27]	UE subdomain v2.0 (only 4 newly added items studied)	General population and patients with musculoskeletal upper extremity disorders	?	Results for comprehensibility and comprehensiveness not reported
			?	3 out of 4 new items (6.5% of item bank) were considered less relevant or describing unusual activities in the Dutch context. Other items of item bank were not studied so no overall conclusion possible
*Structural validity*				
Crins et al. [42]	PF item bank	Dutch adults with chronic pain	+	Sufficient unidimensionality (CFI and TLI = 0.976, RMSEA = 0.122)
			+	Sufficient monotonicity (H ≥ 0.42)
			+	Sufficient local independence**:** 6% of items were flagged, impact negligible (evidence provided)
Crins et al. [43]	PF item bank	Dutch adults receiving physical therapy	+	Sufficient unidimensionality (CFI = 0.924, TLI = 0.923, RMSEA = 0.045)
			+	Sufficient monotonicity (all items except one H ≥ 0.30)
			?	Indeterminate local independence: 8.2% of items were flagged (no statements on impact)
van Bruggen et al. [45]	UE subdomain	Dutch adults with an injury of the upper extremity	−	*Insufficient unidimensionality (CFI* = *0.94, TLI* = *0.93, RMSEA* = *0.10, SRMR* = *0.09)*
				*Local independence, monotonicity and model fit not reported.*^*b*^
Lameijer et al. [46]	UE subdomain	Dutch adults with injury or disorder of upper extremity	+	Sufficient unidimensionality (FA: CFI en TLI = 0.93, RMSEA = 0.099, SRMR = 0.09 (all insufficient), but exploratory bi-factor analysis: ECV 0.68, Omega coefficient 0.80 (sufficient))
			+	Sufficient local independence: 3.3% of items were flagged
			+	Sufficient monotonicity (H = 0.55–0.70)
*Hypotheses testing for construct validity*				
Oude Voshaar et al. [37]	PF item bank	RA patients		*7 out of 8 hypotheses were met* Pearson correlations (with hypothesis):
			+	Age: 0.14 (0.10–0.30)
			+	HAQ-DI: 0.76 (> 0.60)
			+	SF36-PF-10: 0.84 (> 0.60)
				On 10-point numerical rating scales:
			+	Pain: − 0.52 (0.30–0.60)
			+	General health: − 0.53 (0.30–0.60)
			+	Disease activity: − 0.46 (0.30–0.60)
			+	Fatigue: − 0.47 (0.30–0.60)
			−	Stiffness: − 0.63 (0.30–0.60)
				Known-groups validity: no hypothesis
Crins et al. [42]	PF item bank	Dutch adults with chronic pain		*5 out of 6 hypotheses were met*
				Pearson correlations (with hypothesis):
			+	PROMIS pain intensity: − 0.73 (< − 0.70)
			+	NDI: − 0.70 (< − 0.50)
			+	DASH: − 0.86 (< − 0.50)
			+	RMDQ: − 0.70 (< − 0.50)
			+	FIQ: − 0.62 (< − 0.50)
			−	Global health pain: − 0.62 (− 0.50 < r < − 0.30)
Crins et al. [43]	PF item bank	Dutch adults receiving physical therapy		*2 out of 3 hypotheses were met*
			+	Pearson correlations (with hypothesis): SF36-PF10: 0.84 (> 0.70)
			+	HAQ-DI: 0.85 (> 0.60)
			−	Correlation of SF-36-PF10 higher than HAQ-DI: not met
				Total score: 12 out of 15^c^ (80%)
van Bruggen et al. [45]	UE subdomain	Dutch adults with an injury of the upper extremity		*2 out of 3 hypotheses were met* Pearson correlations (with hypothesis):
			+	DASH: − 0.84 (< − 0.50)
			−	PRWE function: − 0.75 (− 0.50 ≤ r ≤ − 0.30)
			+	MHQ-ADL: 0.73 (r ≥ 0.50^d^)
Haan et al. [27]	UE subdomain	Dutch patients with musculoskeletal upper extremity disorders		*4 out of 4 hypotheses were met* Pearson correlations (with hypothesis):
			+	PROMIS pain intensity: − 0.43 (− 0.50 < r ≤ − 0.30)
			+	DASH: − 0.87 (< − 0.50)
			+	FIHOA: − 0.86 (< − 0.50)
			+	MHQ-ADL: 0.87 (> 0.50)
				Total score: 4 out of 5^e^ (80%)
*Cross-cultural validity/measurement invariance*				
Oude Voshaar et al. [41]	PF item bank	RA patients	?	Gender: 5.8% of items (no evidence on impact provided)
			+	Age: 4.1% of items
			+	Language (English): 20.6% of items, impact negligible (evidence provided)
Crins et al. [42]	PF item bank	Dutch adults with chronic pain	+	DIF for gender: none
			+	DIF for age: 0.8% of items
			+	DIF for language (US English): 3.3% of items
Crins et al. [43]	PF item bank	Dutch adults receiving physical therapy	+	DIF for age: 1.7% of items
			?	DIF for gender: 11.6% of items. Claim that impact is negligible, no evidence provided
Crins et al. [44]	PF item bank	Dutch adults with muscoloskeletal pain Dutch adults with osteoarthritis Dutch adults receiving physical therapy Dutch general population	+	DIF between different patient groups: Chronic pain vs. osteoarthritis: 11.6% of items Chronic pain vs. physiotherapy:1.7% of items Chronic Pain vs. general pop.: 1.7% of items Osteoarthritis vs. physiotherapy: 2.5% of items Osteoarthritis vs. general pop.: 11.6% of items Physiotherapy vs. general pop.: 3.3% of items Overall impact negligible (evidence provided)
Haan et al. [27]	UE subdomain	Dutch patients with musculoskeletal upper extremity disorders	+	*DIF for language (English): 17.4% of items*
Lamerijer et al. [46]	UE subdomain	Dutch adults with injury or disorder of upper extremity	+	*Impact negligible (evidence provided).*^*b*^ DIF for age: none
			+	DIF for gender: 2.2%
			+	DIF for duration of complaints: 6.5%
				DIF for language (English): 8.7%
				Impact negligible (evidence provided)
*Internal consistency/measurement precision*				
Oude Voshaar et al. [37]	PF item bank	RA patients	+	“precision is high across all levels of physical functioning” (and results can be assumed to be better than the presented evidence for the PROMIS-PF-20, see Table 6)
Crins et al. [42]	PF item bank	Dutch adults with chronic pain	+	Reliability coefficient > 0.9 between T-scores 28.3–43.1 (1 SD above and below average score)
Crins et al. [43]	PF item bank	Dutch adults receiving physical therapy	+	Reliability coefficient > 0.9 between T-scores 38.8–57.6 (1 SD above and below average score)
van Bruggen et al. [45]	UE subdomain	Dutch adults with an injury of the upper extremity	+	Cronbach’s alpha = 0.98
Lamijer et al. [46]	UE subdomain	Dutch adults with injury or disorder of upper extremity	+	Reliability coefficient > 0.90 for 95.6% of the patient population

CFI, comparative fit index; DASH, Disabilities of the Arm, Shoulder and Hand (subscale disability/symtpoms); DIF, differential item functioning; ECV, explained common variance; FA, factor analysis; FIHOA, Functional Index for Hand Osteoarthritis; FIQ, Fibromyalgia Impact Questionnaire; HAQ-DI, Health Assessment Questionnaire Disability Index; ICF, International Classification of Functioning, Disability and Health; MHQ-ADL, Michigan Hand Outcomes Questionnaire (subscale Activities of Daily Living); NDI, Neck Disability Index; RMDQ, Roland Morris Disability Questionnaire; PROMIS, Patient-Reported Outcomes Measurement Information System; PRWE, patient-rated wrist evaluation; RA, rheumatoid arthritis; RMSEA, root mean square error of approximation; SD, standard deviation; SF36-PF-10, short-form 36 physical functioning scale; SRMR, Standardized Root Mean Squared Error; TLI, Tucker–Lewis index; UE, upper extremity

“ + ” = sufficient, “?” = indeterminate, “-” = insufficient

^a^Evidence for comprehensibility from the general population is considered valid for all other relevant patient populations for this review

^b^Data from Van Bruggen et al. and Haan et al. are also used as part of the larger dataset of Lameijer et al. for partly the same analyses. Only the results from Lameijer are taken into account when the same analyses are conducted

^c^Correlations with the HAQ-DI and SF-36-PF10 were assessed in two studies, but both only counted once for the total score

^d^Adjusted by reviewers to hypothesis of Haan et al. [27] as this was deemed more suitable

^e^Correlations with the DASH and MHQ-ADL were assessed in two studies, but both only counted once for the total score

**Table 6:** Quality of measurement properties per study for the CATs and short forms

References	PROMIS-PF instrument	Population	Score	Description
*Content validity*				
Oude Voshaar et al. [37]	*PROMIS-PF-RA*	RA patients	+	Relevance and comprehensiveness are studied by linking the item bank to the ICF core set for RA: result was good
			?	Comprehensibility not studied
Smit et al. [47]	*PROMIS-PF-GR*	Geriatric rehabilitation patients	+	6 patients and 6 experts interviewed. Relevance, comprehensiveness and comprehensibility good
*Structural validity*				
Chiarotto et al. [36]	*PROMIS-PF-20* *PROMIS-PF-10* *PROMIS-PF-8* *PROMIS-PF-6* *PROMIS-PF-4*	Patients with musculoskeletal pain complaints	+ + + + −/+	Sufficient unidimensionality for all short-forms (CFI and TLI > 0.95) Sufficient local dependence for all short forms (0%-4.7%) Sufficient monotonicity for all short forms Insufficient model fit for the PROMIS-PF-20 (10% of items do not have sufficient fit), sufficient model fit for all other short-forms
Smit et al. [47]	*PROMIS-PF-GR*	Geriatric rehabilitation patients	+	Sufficient unidimensionality (CFI and TLI = 0.95, RMSEA = 0.09. Bifactor analysis: coefficient omega 0.83, and ECV 0.71)
			+	Sufficient local independence: 1.3% of item pairs showed local dependence
			+	Sufficient monotonicity (H = 0.32–0.65)
			?	Model fit not reported
*Hypotheses testing*^*q*^				
Chiarotto et al. [36]	*PROMIS-PF-20* *PROMIS-PF-10* *PROMIS-PF-8* *PROMIS-PF-6* *PROMIS-PF-4*	Patients with musculoskeletal pain complaints	+ + + +	4 out of 4 hypotheses were met for all short forms *Convergent* Pearson correlations (with hypothesis): PROMIS-GH-Physical Health: 0.73–0.76 (≥ 0.60) PROMIS-GH-Mental Health): 0.24–0.27 (0.20–0.50 Pain NRS (− 0.40 < r < − 0.60^a^): − 0.57 < r < − 0.52 *Known-groups* Hypothesis: Patients with chronic pain have worse physical functioning than patients without chronic pain. True for all short-forms
*Cross-cultural validity/measurement invariance*				
Crins et al. [44]	*PROMIS-PF-20*	Patients with muscoloskeletal pain, osteoarthritis, receiving physical therapy and general population	+	DIF between patient groups: 15% of items were flagged in total across all patient group comparisons. Impact negligible. (evidence provided)
Smit et al. [47]	*PROMIS-PF-GR*	Geriatric rehabilitation patients	?	DIF compared to general population: 20.8% of items. Authors state that impact on total score is negligible (no evidence provided)
*Internal consistency/measurement precision*				
Oude Voshaar et al. [37]	*PROMIS-PF-RA*	RA patients	+	Reliability coefficient > 0.9 (test information 10) between theta − 2.2 and 0.8
Crins et al. [42]		Dutch adults with chronic pain		Between T-scores 28.3–43.1 (1 SD above and below average score):
	*PROMIS-PF-20*		+	Reliability coefficient > 0.9
	*PROMIS-PF-10*		−	Reliability coefficient < 0.9
	*CAT-20*		+	Reliability coefficient > 0.9
	*CAT-10*		+	Reliability coefficient > 0.9
	*CAT-8*		−	Reliability coefficient < 0.9
	*CAT-6*		−	Reliability coefficient < 0.9
	*CAT-4*		−	Reliability coefficient < 0.9
Crins et al. [43]		Dutch adults receiving physical therapy		Between T-scores 38.8–57.6 (1 SD above and below average score):
	*PROMIS-PF-20*		+	Reliability coefficient > 0.9
	*PROMIS-PF-10*		−	Reliability coefficient < 0.9
	*PROMIS-PF-8*		−	Reliability coefficient < 0.9
	*PROMIS-PF-6*		−	Reliability coefficient < 0.9
	*PROMIS-PF-4*		−	Reliability coefficient < 0.9
	*CAT-20*		+	Reliability coefficient > 0.9
	*CAT-10*		+	Reliability coefficient > 0.9
	*CAT-8*		+	Reliability coefficient > 0.9
	*CAT-6*		+	Reliability coefficient > 0.9
	*CAT-4*		−	Reliability coefficient < 0.9
Chiarotto et al. [36]		Patients with muskoskeletal pain complaints		Between theta − 2.2 and − 0.8 (1 SD above and below average score in study population):
	*PROMIS-PF-20*		+	Reliability coefficient > 0.9
	*PROMIS-PF-10*		+	Reliability coefficient > 0.9
	*PROMIS-PF-8*		+	Reliability coefficient > 0.9
	*PROMIS-PF-6*		+	Reliability coefficient > 0.9
	*PROMIS-PF-4*		+	Reliability coefficient > 0.9
van Bruggen et al. [45]	*PROMIS-UE-7a*	Dutch adults with an injury of the upper extremity	+	Cronbach’s alpha = 0.90
Lamerijer et al. [46]	*PROMIS-UE-7a*	Dutch adults with injury or disorder of upper extremity	+	For 88.3% of patient pop. reliability coefficient > 0.90
	*UE CAT*^*b*^		+	For 91.1% of patient pop. reliability coefficient > 0.90
	*UE CAT-7*		+	For 87.4% of patient pop. reliability coefficient > 0.90
Smit et al. [47]	*PROMIS-PF-GR*	Geriatric rehabilitation patients	?^c^	Cronbach’s alpha = 0.94

CAT-[number], computer adaptive test with fixed [number] of items; CFI, comparative fit index; DIF, differential item functioning; ECV, explained common variance; FA, factor analysis; GR, geriatric rehabilitation; ICF, International Classification of Functioning, Disability and Health; IRC, item response curve; NRS, numeric rating scale; PROMIS, Patient-Reported Outcomes Measurement Information System; PROMIS-GH, PROMIS general health; PROMIS-PF-[number], short form with [number] of items; RA, rheumatoid arthritis; RMSEA, root mean square error of approximation; SD, standard deviation; TLI, Tucker–Lewis index; UE, upper extremity

^a^Adjusted hypothesis, original hypothesis was positive (above 0)

^b^With standard PROMIS CAT stopping rules (standard error of 3 on the T-score metric is reached, maximum of 12 items)

^c^Requirement of at least low quality evidence for structural validity not met

**Table 7:** Summary of the evidence per item bank/instrument

Item bank/instrument	Validity				Reliability		Responsiveness
	Content validity	Structural validity	Construct validity via hypothesis testing	Cross-cult. validity/measurement invariance	Internal consistency/measurement precision	Reliability/measurement error	Responsiveness
	Comprehensibility/relevance/comprehensiveness						
*PF item bank*	+^a^/./.	+++	+++	+	+++	.	.
	+^b^/+^c^/+						
PROMIS-PF-20	+^b^/+^c^/.	−	+	+	++^c^	.	.
PROMIS-PF-10	+^b^/+^c^/.	+	+	.	−	.	.
PROMIS-PF-8	+^b^/+^c^/.	+	+	.	±	.	.
PROMIS-PF-6	+^b^/+^c^/.	+	+	.	±	.	.
PROMIS-PF-4	+^b^/+^c^/.	+	+	.	±	.	.
PROMIS-PF-RA	+^b^/+/+	.	.	.	+	.	.
PROMIS-PF-GR	+/+/+	?	.	?	?	.	.
CAT-20	+^b^/+^c^/.	.	.	.	++	.	.
CAT-10	+^b^/+^c^/.	.	.	.	++	.	.
CAT-8	+^b^/+^c^/.	.	.	.	−−^d^	.	.
					+^e^		
CAT-6	+^b^/+^c^/.	.	.	.	−−^d^	.	.
					+^e^		
CAT-4	+^b^/+^c^/.	.	.	.	−−	.	.
*UE subdomain*	./?^f^/.	+++	.	+	+++	.	.
PROMIS-UE-7a	./−^g^/.	.	.	.	+	.	.
UE CAT^h^	././.	.	.	.	+	.	.
UE CAT-7	././.	.	.	.	+	.	.

CAT-[number], computer adaptive test with fixed [number] of items; GR, geriatric rehabilitation; PROMIS, Patient-Reported Outcomes Measurement Information System; PROMIS-PF-[number], short form with [number] of items; RA, rheumatoid arthritis; UE, upper extremity

(+)/(− −) = very low quality evidence of sufficient or insufficient quality of the measurement property

±  = low quality evidence of sufficient or insufficient quality of the measurement property

++/– = moderate quality evidence of sufficient or insufficient quality of the measurement property

+ + +/— = high quality evidence of sufficient or insufficient quality of the measurement property

? = indeterminate evidence

. = not studied

^a^Sufficient comprehensibility in general population; relevance and comprehensiveness not studied

^b^For patients with RA. Evidence for comprehensibility of the Dutch–Flemish PROMIS-PF item bank from the general population is considered valid for all other relevant patient populations and for all instruments derived from this item bank. for this review

^c^For patients with RA (not studied in other populations). We have extrapolated the results from the PROMIS-PF item bank to the instruments derived from the item bank

^c^Downgrade of the evidence due to inconsistent results for which there was no potential explanation

^d^For patients with pain. (Split up due to inconsistent results that potentially be explained by the study population.)

^e^For physical therapy patients. (Split up due to inconsistent results that potentially be explained by the study population.)

^f^Not studied for comprehensibility and comprehensiveness. Only four items of item bank studied for relevance, therefore indeterminate evidence for overall relevance of item bank

^g^Derived from the study of the full item bank: 2/7 (29%) of items are considered less relevant in a Dutch population

^h^ With standard PROMIS CAT stopping rules (standard error of ≤ 3 on the T-score metric is reached, maximum of 12 items)

#### Item banks

##### Validity

Aspects of content validity of the PROMIS-PF item bank were evaluated in two studies. The study of Terwee et al. [24] describes the translation of the item bank, which was done according to the strict standards of PROMIS. The study also included a cognitive validation study, in which the comprehensibility of the item bank was studied. After adaptation of some items this was found to be sufficient. The study of Oude Voshaar et al. [37] studied the content validity of the item bank for patients with RA. It linked the items of the PROMIS-PF item bank to the International Classification of Functioning, Disability and Health (ICF) core set for patients with RA using proposed linking rules [49, 50] to study relevance and comprehensiveness. The PROMIS-PF item bank was shown to comprehensively reflect nearly all aspects of physical function needed to represent the experience of patients with RA and to contain only relevant items. The two assessments with doubtful methodological quality result in low quality evidence for sufficient comprehensibility of the item bank in general and for sufficient content validity in patients with RA.

Aspects of content validity of the UE subdomain were only reported in the study of Haan et al. [27]. They translated the four new items that were added when v1.2 of the UE subdomain was developed into v2.0 and performed a cognitive validation study. They found that three of the four new items were less relevant or less common activities in a Dutch context (6.5% of item bank), but since the other items of the item bank were not studied it was not possible to determine if at least 85% of items is relevant based on this study. Results regarding comprehensibility are not reported. This assessment with doubtful methodological quality provides indeterminate evidence for content validity of the UE subdomain.

**Structural validity** of the PROMIS-PF item bank was assessed in two studies [42, 43]. All aspects of structural validity (unidimensionality, monotonicity and local dependence) were found to be sufficient. Some local dependence was found in both studies, but one (high quality) study provided evidence that the impact was negligible. This results in high quality evidence for sufficient structural validity.

Structural validity of the UE subdomain was assessed in two studies [45, 46]. The study of Lameijer et al. partially used the same data as the other study, therefore only the former study (with a larger sample and more analyses) was used to determine the evidence. There is high quality evidence for sufficient unidimensionality of the UE subdomain, and moderate quality evidence (downgraded for sample size) for sufficient structural validity as a whole.

**Construct validity via hypothesis testing** was assessed in three studies for the PROMIS-PF item bank [35–37] and in two studies for the UE subdomain [27, 45]. For convergent validity and known-groups validity together, 12 out of 15 hypotheses (80%) for unique correlations/group differences were correct for the PF item bank, and 4 out of 5 (80%) for the UE subdomain. Correlations for some instruments (i.e. HAQ-DI, SF-36-PF10 and MHQ-ADL) were determined in more than one study. Since these showed consistent positive results in study populations of adequate sample size, even without statistical pooling these correlations clearly confirmed the hypothesis and contributed to the high quality evidence for sufficient construct validity for both the PROMIS-PF item bank and the UE subdomain.

**Cross-cultural validity/measurement invariance** was assessed in four studies for the PROMIS-PF item bank [41–44]. Two studies assessed DIF for language, with different results: 3.3% of items showed DIF in chronic pain patients, and 20.6% in patients with RA. However, since the latter study provided evidence that the DIF has negligible impact, the overall conclusion is that there is low quality evidence for sufficient cross-cultural validity. Two studies showed almost no DIF for age, while for gender one study showed no DIF and two studies found more than 5% DIF without showing evidence of the impact (leading to indeterminate results). The overall conclusion is low quality evidence for sufficient measurement invariance.

Two studies assessed the cross-cultural validity/measurement invariance for the UE subdomain [27, 46], both with doubtful methodological quality, but as with structural validity only the study of Lameijer et al. was taken into account. Some DIF was found but evidence showed this to be negligible. Therefore, there is low quality evidence for sufficient cross-cultural validity and measurement invariance for the UE subdomain.

##### Reliability

**Internal consistency/measurement precision** was evaluated in three studies for the PROMIS-PF item bank [37, 42, 43]. All studies of the PROMIS-PF item bank showed a reliability coefficient of > 0.9 between two standard deviations around the average theta for the study population. Two studies assessed internal consistency/measurement precision for the UE subdomain [45, 46]. The study of van Bruggen et al. determined Cronbach’s alpha, which provides only very low quality evidence as this is not the preferred parameter for IRT-based scores. However, the high quality study of Lameijer et al. showed an adequate measurement precision on the underlying metric. For both the PROMIS-PF item bank and the UE subdomain there is high quality evidence for sufficient internal consistency/measurement precision.

#### Instruments

##### Validity

**Content validity** was only studied for two newly developed short forms, in two studies of doubtful quality [37, 47]. The PROMIS-PF-RA, aimed at patients with RA, consists of items that each match one different aspect of the ICF core set for RA (described in “Item banks” section). The PROMIS-PF-GR, aimed at geriatric rehabilitation patients, was developed with the help of experts in the field of geriatric rehabilitation. Its content validity was further confirmed by interviews with patients. For both short forms, there is low quality evidence for sufficient content validity.

Furthermore, results on the comprehensibility and relevance of the items in the PROMIS-PF short forms and PROMIS-PF CATs was extrapolated from the results of the PROMIS-PF item bank. This results in low quality evidence for the comprehensibility of standard short forms and CATs, and low quality evidence for their relevance in patients with RA. There is no evidence for the comprehensiveness of these instruments.

**Structural validity** was studied and found sufficient for all standard short forms except the PROMIS-PF-20 in one study of doubtful methodological quality [36]. Therefore, there is low quality evidence for sufficient structural validity of the PROMIS-PF-10, -8, -6, and -4. For the PROMIS-PF-20, the model fit was insufficient in 2 of the items, resulting in low quality evidence for insufficient structural validity. Structural validity was also assed in one study for the PROMIS-PF-GR [47], which found positive results for unidimensionality, monotonicity and local dependence, but the sample was considered too small for the IRT analyses. Furthermore, model fit is not reported. Therefore, even though there is high quality evidence for sufficient unidimensionality (studied with FA), there is indeterminate evidence for structural validity as a whole.

**Construct validity via hypothesis testing** was assessed in only one study, of doubtful methodological quality, for the standard short forms [36]. The four hypotheses were all met. This results in low quality evidence for sufficient construct validity of the standard short forms.

**Cross-cultural validity** was not studied for the short forms and CATs, but there were two studies that assessed measurement invariance of the PROMIS-PF-20 [44] and the PROMIS-PF-GR [47], respectively. It was found that 15% of the items of the PROMIS-PF-20 were flagged across all analyses for DIF between patient groups/the general population. Evidence was provided that the impact was negligible. DIF for age and gender was not studied. Therefore, there is low quality evidence for sufficient measurement invariance between patient groups of the PROMIS-PF-20. For the PROMIS-PF-GR, evidence is indeterminate because 20.8% of items showed DIF compared to the general population and no evidence was provided on the impact on item parameters or ability estimates.

##### Reliability

**Internal consistency/measurement precision** was the most frequently assessed measurement property for the PROMIS-PF instruments. Standard PROMIS short forms were evaluated in three studies [36, 42, 43], CATs with different fixed numbers of items in two of those. For the PROMIS-PF-20, CAT-20, and CAT-10, there is moderate quality evidence for sufficient measurement precision.

For the PROMIS-PF-10 results were deemed insufficient with the quality of evidence downgraded to “low” because of inconsistent results across studies (two studies showing insufficient and one study showing sufficient measurement precision). For PROMIS-PF-8, -6, -4, and CAT-4, results were scored as ‘inconsistent’ because there were only two studies for these instruments and they showed different results. For the CAT-8 and CAT-6 there are also inconsistent results, but these can possibly be explained by the study population: for pain patients (with relatively low scores) there is moderate quality evidence for insufficient measurement precision, while for physical therapy patients (with scores closer to the average in the general population) there is low quality evidence for sufficient measurement precision.

Regarding the newly developed short forms, for the PROMIS-PF-RA there is low quality evidence for sufficient measurement precision. The evidence for the PROMIS-PF-GR is indeterminate due to insufficient evidence on structural validity. Furthermore, there is low quality evidence for sufficient measurement precision for the PROMIS-UE-7a, and the UE CATs.

## Discussion

This systematic review shows evidence for sufficient structural validity, measurement precision, construct validity, and cross-cultural validity of the Dutch–Flemish PROMIS-PF item bank v1.2. For the UE subdomain item bank there is high quality evidence for sufficient structural validity and measurement precision. Content validity of these item banks has not been thoroughly demonstrated in a Dutch–Flemish context. All instruments that are based on these item banks have far less robust evidence: there are fewer validation studies available and the studies are of lower methodological quality. Additionally, test–retest reliability and responsiveness have so far not been studied.

Content validity is considered the most important measurement property by the COSMIN team, because first of all it should be clear that the items of the PROM are relevant, comprehensive, and comprehensible with respect to the construct of interest and target population [28].

To what extent *comprehensiveness* can be clearly determined for PROMIS item banks and CATs is a point of discussion. First of all, comprehensiveness may be hard to achieve for an item bank that is aimed at being suitable for all (patient) populations. For example, the study of Oude Voshaar et al. [37] concluded that there were three relevant aspects of physical functioning in patients with RA that were not covered in the PROMIS-PF item bank (e.g. moving around with equipment). In this review, this was still considered sufficient comprehensibility, but this is up for debate. Secondly, sufficient comprehensiveness of a complete item bank does not mean that the derived instruments (with which the actual measurements take place) are necessarily comprehensive. While for short forms this can be evaluated, this is challenging for CATs in which different items can be provided in each individual administration [51]. This might by definition imply a potential lack of content validity of CATs, unless it is decided that comprehensiveness is not a strict criterion. Comprehensiveness of a CAT could however be improved by ‘content balancing’ in which it is ensured that in each CAT items of each subdomain of the construct are presented [52]. In the case of PROMIS-PF item bank this could be the upper extremity, lower extremity, and central regions (neck, back) domains. However, this has so far not been applied for PROMIS-PF instruments.

In contrast to comprehensiveness, *relevance* of the items may generally be higher when using CATs, as the presented items are more tailored to an individual’s level of physical functioning. More discussion should take place in the scientific community on the definition of content validity for CATs and possible ways to establish this for CATs.

In addition, one might question the need to evaluate comprehensibility and relevance separately for each language and each patient group in each country. Studying the relevance of items in a new culture is definitely relevant: for example, the study of Haan et al. [27] shows that three out of four newly added items for the UE subdomain v2 were less relevant for the Dutch population. However, it may be possible to extrapolate results on relevance and comprehensiveness for different patient populations from other languages. The development and content validity of the PROMIS-PF short forms have been studied previously in a systematic review by Chiarotto et al. from 2018 [53] without language restrictions. The included studies provided information on the development of the short forms only. The review found very low quality evidence for the sufficient content validity of the PROMIS-PF-20 and -10, while for the other short forms content validity was insufficient due to lack of comprehensiveness. This, therefore, underlines the necessity for more content validity studies of the PROMIS-PF instruments, both for the original US English version and the Dutch–Flemish version.

Not all results for the measurement properties were described or shown clearly in the studies. For example, measurement precision of the item bank is described in the study of Oude Voshaar et al. as “high across all levels of physical functioning” with no further evidence provided. Statements about the negligible impact of DIF are also regularly not supported by evidence in the article. These instances were discussed in the review team and judged on a per-case basis and our conclusion is reported in the evidence tables.

The COSMIN criteria [28, 31] do not include all necessary specific criteria for IRT-based instruments, and no specific (adapted) criteria for item banks are present. Therefore, for some measurement properties, we created additional criteria based on consensus in the review team. There is no one right way to set these criteria, and other review teams might make different choices. For example, our criterion for measurement precision could be up for discussion both considering the cut-off for the reliability coefficient (≥ 0.90 is a strict criterion, based on patient-level use) and considering the range of theta along which this minimal reliability coefficient should be reached. Using different criteria might result in different conclusions about the quality of some measurement properties. One of the included studies used a much stricter criterion than we did [36] (reliability coefficient ≥ 0.90 between theta − 4 and 4). If we had used this criterion this would have led to the conclusion that all instruments have insufficient measurement precision, and for the item bank as a whole there would be inconsistent results. On the other hand, some might argue to lower the criterion to reliability coefficient ≥ 0.80. In this case, more of the briefer PROMIS-PF instruments would have shown sufficient results for measurement precision at a range of 2 SD around the average (i.e. PROMIS-PF-8, CAT-8, -6 and -4). However, considering the proposed use of PROMIS in Dutch clinical practice we consider our relatively strict criterion the most relevant for this review.

Additionally, we used different criteria than the ones COSMIN suggest for local dependence (part of structural validity) and cross-cultural validity/measurement invariance. According to COSMIN, no local dependence or DIF should be allowed, whereas we considered a proportion of items with significant local dependence or DIF ≤ 5% (i.e. the proportion of items that could be flagged by chance) as acceptable. We also considered it acceptable if DIF was present in > 5% of items but evidence was provided that this had no impact item parameters or ability estimates. In case of local dependence, the strict COSMIN criterion would have led to the conclusion that the item bank as a whole and the PROMIS-PF-20 show insufficient local dependence and therefore insufficient structural validity. The other short forms do no show local dependence [36]. In the case of cross-cultural validity/measurement invariance, this would have resulted in insufficient results for the PROMIS-PF item bank and the UE subdomain as well as the short forms for which this was studied. However, we believe these criteria are too strict, certainly for item banks but also for instruments with larger numbers of items.

The COSMIN risk of bias checklist might be too “one size fits all” for the study quality of some measurement properties. The checklist understandably includes an item regarding necessary similarity of the groups when studying cross-cultural validity (i.e. the Dutch and the US study population) or measurement invariance. In practice, however, most studies did not compare groups that (apart from the group difference under study) were similar, or they did not report on this clearly. This resulted in lower scores for methodological quality and the quality of the evidence. However, one could argue that when different populations are compared, DIF could be caused by both the group difference under study and other differences. Therefore, if results are still sufficient despite this methodological flaw, it could actually be considered good evidence for sufficient cross-cultural validity. Keeping this in mind, the evidence for sufficient cross-cultural validity might be considered high quality.

There are some clear gaps in the evidence of PROMIS-PF and the UE subdomain and their related instruments. First of all, evidence for the instruments is still far less robust than evidence for the item banks. While evidence for sufficient measurement properties of the item banks is an important first step, the instruments will have to prove their worth in actually measuring patient outcomes. In this context, it is also important to evaluate actual performance of true CATs in practice rather than simulated CATs, and to administer and evaluate the short forms as such. This would also allow for the CATs to be tested on criterion validity, with the full item bank as the gold standard. Furthermore, and especially for the instruments, studies showing sufficient evidence for content validity (especially relevance and comprehensiveness), test–retest reliability and responsiveness are essential to be able to conclude that these instruments are suitable for their proposed uses in research and clinical practice. Lastly, except for the translation study, all studies were conducted in Dutch populations only. An additional study in the Flemish population would have added benefit for drawing conclusions about the quality of measurement properties of the Dutch–Flemish item bank in both cultures.

With respect to PROMIS-PF, more research into CATs and their application would be a useful next step. As mentioned above, the exploration of content balancing may be interesting in order to ensure content validity of the PROMIS-PF CATs. Additionally, more research into the measurement precision achieved with different numbers of items could provide more insight into selecting the optimal number of items in a PROMIS-PF CAT.

More generally, the field would benefit from more consensus on suitable methods and criteria for evaluating item banks and CATs with IRT methods. Currently, each review on this topic will have to consider their own criteria, which does not benefit comparability. Guidelines on suitable methods and criteria could also be used by researchers conducting and publishing future validation studies, potentially improving both methodology and consistency of reporting in the published articles. There could potentially be a role for the COSMIN initiative here, as they have experience with this process for CTT methods.

### Strengths and limitations

The strengths of this study are the systematic use of the COSMIN checklist to evaluate the quality of the included studies, and the critical assessment of the criteria for good measurement properties. Furthermore, a strength of this review is the critical approach towards the criteria for IRT instruments/item banks where necessary. However, this is at the same time a limitation: as discussed above, different choices regarding these criteria can be made by different review teams. Furthermore, only studies into the measurement properties of the Dutch PROMIS-PF item bank were taken into account in this review, while international studies may provide indirect evidence for some measurement properties of the Dutch–Flemish PROMIS-PF as well.

## Conclusions

The first studies into the Dutch–Flemish PROMIS-PF item bank and the UE subdomain show promising results, with especially high quality evidence for structural validity and measurement precision. However, more studies, and with higher methodological quality, are needed to study the instruments derived from these item banks that are used to actually measure patient outcomes. These studies should also evaluate content validity, reliability and responsiveness, measurement properties which have not been (sufficiently) studied. Consensus in the field about the most suitable methods and criteria for studies into IRT instruments would benefit future research.

## Supplementary Information

**Additional file 1.** The Pubmed search strategy used for this review.

## Data Availability

Data sharing is not applicable to this article as no datasets were generated or analysed during the current study.
